# Characterization of polyphenols and carbohydrates exuded by *Phaeodactylum tricornutum* diatom grown under Cu stress

**DOI:** 10.1038/s41598-024-60252-x

**Published:** 2024-04-23

**Authors:** Milagros Rico, Paula Santiago-Díaz, Argimiro Rivero, Juana Magdalena Santana-Casiano

**Affiliations:** 1 Departamento de Química , Facultad de Ciencias del Mar, Universidad de Las Palmas de Gran Canaria, Campus de Tafira, 35017 Las Palmas de Gran Canaria, Canary Islands Spain; 2 Instituto de Oceanografía y Cambio Global (IOCAG), Universidad de Las Palmas de Gran Canaria, Unidad Asociada ULPGC‐CSIC, Las Palmas de Gran Canaria, Spain

**Keywords:** Environmental sciences, Ocean sciences

## Abstract

This study is focused on analysing polyphenols and carbohydrates released by *Phaeodactylum tricornutum* (*P. tricornutum*) diatoms cultured in natural seawater enriched with sublethal and lethal Cu doses. Cu concentrations of 0.31, 0.79 and 1.57 µM reduced cell densities by 37, 82 and 91%, respectively, compared to the control. The total sum of all identified polyphenols and total carbohydrates released by cells grown under lethal Cu levels increased up to 18.8 and 107.4 times, respectively, compared to data from a control experiment. Four different in vitro assays were used to estimate the antioxidant activities of the extracellular compounds: 2,2-diphenyl-1-picrylhydrazyl (DPPH) radical inhibition, cupric ion reducing antioxidant capacity (CUPRAC), ferric reducing antioxidant power and Cu complexing ability (CCA). The highest antioxidant activities were observed in the Cu lethal treatments, where the CCA assay exhibited a greater increase (up to 32.2 times higher than that found in the control experiment) to reduce the concentration of free Cu in the medium and its toxicity. The presence of Cu stimulated the release of polyphenols and carbohydrates to the medium as a detoxification mechanism to survive under lethal levels of Cu regulating its speciation.

## Introduction

Marine microorganisms are known to release organic ligands that can regulate and modify the speciation of trace metals in the surrounding environment, affecting the bioavailability of these trace metals needed in a vast array of enzymatic reactions, and their potential toxicity^1–3^. Thus, chemical speciation may aid biosorption capacity and cellular uptake of metals in regions of low concentration and hinder toxicity in the presence of high concentration^4^. Relatively little is known about the chemical characteristics of extracellular organic compounds present in seawater due to their complex chemical structures and molecular sizes, and their low concentration^5,6^. These organic ligands bind more than 99.6% of the total iron and copper in natural waters^7^ and include a wide range of organic compounds such as proteins, lipids, carbohydrates, uronic acids and polyphenols^8–10^.

Copper is an essential nutrient that can become toxic at certain concentrations and exhibits affinity for a wide range of ligands containing sulfur, nitrogen and oxygen^11,12^. The level of this trace metal varies from 0.008 to 0.05 μM in natural environments and is increased by local anthropogenic activities or natural activities such as volcanic episodes up to 3.0 μM^13–15^. The presence of Cu in coastal seawater at high concentrations has a direct impact on marine microorganisms, causing serious damage, such as disruption of important proteins or alteration of the oxidative balance of cells, modifying the composition of the extracellular matter in the surrounding medium^16,17^. As a consequence, Cu toxicity induces mortality in phytoplankton, modifies the start and end timing of blooms and their amplitude, changes the phytoplankton community structure and affects the survival of important species, contributing to biodiversity loss^18,19^.

The role of extracellular organic matter exuded by marine phytoplankton exposed to toxic levels of Cu is not entirely clear. Previous research reported defence strategies of *Phaeodactylum tricornutum* (*P. tricornutum*) diatom that consist of exuding various types of metabolites^20^. Determining how the concentrations of extracellular substances secreted by phytoplankton change in response to elevated levels of Cu could provide a better understanding of their role with respect to regulatory mechanisms for metals that are potentially toxic, and could be useful in helping to explain the metal bioavailability.

The presence of strong Cu-binding ligands in surface seawaters stabilizes the excess of dissolved copper^21^. Carbohydrates and phenolic compounds can influence metal ion chemistry and bioavailability^2,22^. Catechin, sinapic acid and gallic acid were found to increase the persistence of dissolved Fe, regenerating Fe(II) in seawater from 0.05 to 11.92%^23^. Furthermore, iron bioavailability also increased when three different saccharides were used in cultured and natural eukaryotic phytoplankton populations, suggesting that this is a generalizable phenomenon^24^.

The aim of this work was to evaluate the content of polyphenols and carbohydrates released by the diatom *P. tricornutum* grown for 12 and 18 days in natural seawater as a control, and in seawater enriched with three copper concentrations lower than those found in polluted coastal areas^13,15^, two lethal and one sublethal (1.57, 0.79 and 0.31 µM, respectively). The effects of Cu toxicity on the growth and intracellular productivity of these diatoms have been previously reported^25^. The present study is focused on the change in the characterization of both polyphenols and carbohydrates exuded by the diatoms grown under the same conditions in cultures, where the only variable modified was the copper concentration. Therefore, the total extracellular carbohydrates were evaluated by using the phenol–sulfuric acid reaction^26^, and 10 selected phenolic compounds were identified and quantified by reversed-phase high-performance liquid chromatography (RP-HPLC) due to their Cu chelating ability^27–29^. In addition, the antioxidant activities of the organic compounds isolated by solid phase extraction (SPE) from seawater samples enriched with exudates were determined through four different tests: free radical scavenging ability (RSA) against DPPH radical; FRAP, CUPRAC and CCA assays^30^.

## Results

### Cell growth and productivity under Cu pressure

**Figure 1:**
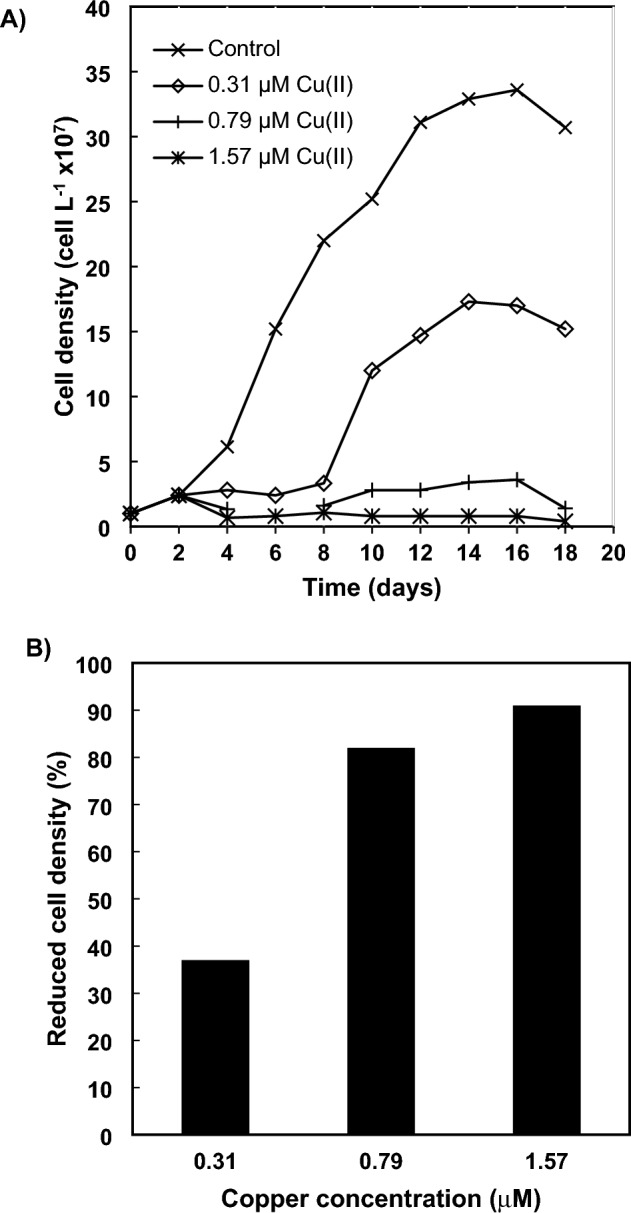
Cell density of *P. tricornutum* grown under different Cu(II) concentrations: (**A**) Cell growth curves; (**B**) Decrease in cell density.

High concentrations of metals such as copper induce stress in phytoplankton that changes their growth patterns as well as their interaction with the environment. Figure 1 summarizes the effects of Cu toxicity on *P. tricornutum* diatom growth, previously reported by Santiago-Díaz et al.^25^ In brief, a reduction in both the growth curve and the biomass generated was observed. The mean absolute growth rate was 2.11 × 10^7^ cell L^−1^ day^−1^ achieving a maximum cell density of 33.6 × 10^7 ^cell L^−1^ during the stationary phase, which ended after 16 days. Considering the control culture as a reference to compare the organic compounds released into the environment under different copper concentrations, the analysis was performed after 12 days (in the exponential phase before entering stationary phase) and after 18 days (in the dead phase).

A Cu concentration of 0.31 µM was a sublethal dose for the microalga, with a biomass reduction of 37% compared to the control. A lack of an exponential phase of growth and cell density decreases of 82 and 91% were observed at the highest Cu concentrations (0.79 and 1.57 µM, respectively), indicating that these Cu levels were lethal for *P. tricornutum* under our experimental conditions. A more detailed description is given by Santiago-Díaz et al.^25^.

**Figure 2:**
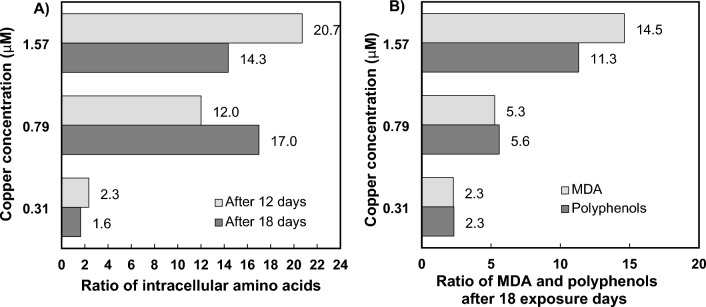
Ratio of intracellular amino acids (**A**), polyphenols (**B**) and MDA (**B**) in *P. tricornutum* diatoms exposed to Cu compared to those in control cells (data extracted from Santiago-Díaz et al., 2023).

Santiago-Díaz et al.^25^ reported the accumulation of intracellular free amino acids and polyphenols after 18 days of exposure to lethal levels of Cu (Table 1). Under these conditions, the total sum of all polyphenols identified in the cells increased up to 11.3 times compared to the control (Fig. 2), and the highest antioxidant activities were also observed in cells exposed to lethal Cu treatments. The production of malondialdehyde (MDA), commonly caused by an increase in free radicals^31^, evidenced enhanced oxidative stress in cells cultured under lethal Cu levels by extending its concentration up to 14.5-fold, and presented a linear correlation (r = 0.9999; *p* < 0.05) with the production of phenolic compounds (Fig. 2).

**Table 1:** Productivity data of *P. tricornutum* cells exposed to different Cu concentrations (extracted from Santiago-Díaz et al.^25^).

	Control	Cu(II) 0.31 μM	Cu(II) 0.79 μM	Cu(II) 1.57 μM
Total sum of the identified intracellular amino acids^a^				
After 12 days	10.11 ± 0.62	23.71 ± 0.89	121.40 ± 3.13	209.40 ± 9.45
After 18 days	7.83 ± 0.24	16.6 ± 0.3	171.8 ± 5.7	145.0 ± 6.1
Total sum of identified intracellular phenolics described in Section “Cell growth and productivity under Cu pressure”^b^				
After 18 days	63.9 ± 15.5	148.4 ± 38.7	357.2 ± 23.4	723.2 ± 55.0
Intracellular MDA contents^b^				
After 18 days	4.5 ± 0.4	10.25 ± 0.07	23.7 ± 0.1	65.8 ± 0

^a^Results expressed as fmol of amino acid cell^−1^.

^b^Results expressed as amol cell^−1^.

All results are expressed as means ± standard deviations of three measurements.

### Characterization of phenolic compounds in exudates

**Figure 3:**
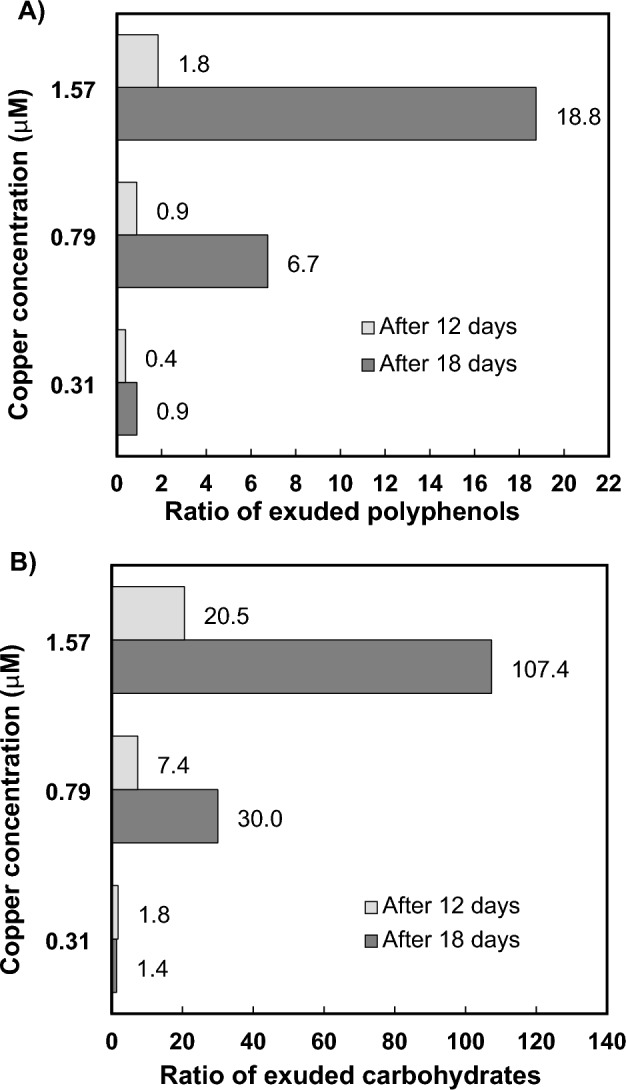
Ratio of exuded polyphenols (**A**) and carbohydrates (**B**) by *P. tricornutum* diatoms exposed to Cu for 12 and 18 days compared to those released by control cells.

The results of the identification and quantification of 10 different phenolics in *P. tricornutum* exudates are summarized in Table 2. After 12 days, the total amount of all phenolic compounds identified was lower than that of control cells at the sublethal dose of Cu, was similar at the lowest lethal dose, and was higher under the highest Cu level. After 18 days, the total phenolic content remained similar at the sublethal Cu dose and was 6.7- and 18.8- fold higher at the lethal Cu levels compared with the control experiment (Fig. 3). However, longer culture periods reduced exuded phenolics from 71.0 (after 12 days) to 30.84 (after 18 days) in the control experiment and increased them under lethal Cu concentrations.

**Table 2:** Phenolic compounds exuded by the diatom *P. tricornutum* grown under different copper concentrations and exposure times.

Phenolic compound	Control	Cu(II) 0.31 µM	Cu(II) 0.79 µM	Cu(II) 1.57 µM
After Cu exposure periods of 12 days				
GAL	4.57 ± 0.74	< LOQ	< LOQ	< LOQ
PCA	10.7 ± 2.8	< LOQ	2.33 ± 0.01	< LOQ
SYR	30.9 ± 1.9	1.92 ± 0.98	41.27 ± 0.79	107.32 ± 2.20
ECAT	20.9 ± 0.46	3.60 ± 0.40	19.77 ± 6.04	23.41 ± 7.85
RU	3.9 ± 0.7	22.30 ± 4.39	< LOQ	< LOQ
Sum	71.0 ± 6.6	27.81 ± 5.77	63.37 ± 6.84	130.72 ± 10.04
After Cu exposure periods of 18 days				
PCA	< LOQ	< LOQ	< LOQ	15.07 ± 1.86
CAT	< LOQ	2.43 ± 0.41	< LOQ	< LOQ
VAN	1.31 ± 0.08	< LOQ	< LOQ	< LOQ
SYR	19.22 ± 0.39	15.81 ± 0.31	101.33 ± 3.43	335.08 ± 0.06
ECAT	10.12 ± 0.21	7.85 ± 0.99	106.77 ± 32.31	228.16 ± 11.74
RU	0.19 ± 0.01	1.53 ± 0.40	< LOQ	< LOQ
Sum	30.84 ± 0.69	27.62 ± 2.11	208.09 ± 35.75	578.31 ± 13.66

The results are expressed as attomol cell^−1^ (means ± standard deviations of three measurements).

SYR and ECAT were found in all the experiments at maximal concentrations compared to the other phenolic compounds tested. SYR content was 3.5 and 17.4 times higher than that of the control (after 12 and 18 days, respectively). GA, COU and FA were below the limit of quantification (LOQ) in all diatom exudates.

### Total carbohydrates

Table 3 summarizes the carbohydrates released by diatoms after a culture period of 12 and 18 days. Compared with control cells, extracellular carbohydrates levels increased with the addition of Cu to the medium after both culture periods, being up to 20.5- and 107.4-fold higher in cells grown under lethal levels of Cu after 12 and 18 days, respectively (Fig. 3). However, the level of carbohydrates was higher after 12 days (0.57 pmol Glc eq cell^−1^) than after 18 days in the control experiment (0.34 pmol Glc eq cell^−1^). The same behavior was observed in the Cu sublethal enrichment assay, whereas cells exposed to lethal levels of Cu showed a higher content of extracellular carbohydrates after 18 days of culture periods than those quantified after 12 days (Table 3).

**Table 3:** Total carbohydrates exuded by *P. tricornutum* diatom grown in seawater enriched with different copper concentrations and incubated for 12 and 18 days.

Time (days)	Control	Cu(II) 0.31 µM	Cu(II) 0.79 µM	Cu(II) 1.57 µM
12	0.57 ± 0.04	1.03 ± 0.02	4.2 ± 0.2	11.7 ± 0.6
18	0.34 ± 0.06	0.47 ± 0.03	10.2 ± 1.0	36.5 ± 2

The results are expressed as pmol Glc eq cell^−1^ (means ± standard deviations of three measurements).

### Antioxidant capacity assays

Table 4 shows the antioxidant activities of *P. tricornutum* exudates analysed by DPPH, FRAP, CUPRAC and CCA assays after Cu exposure for 18 days. An enhanced antioxidant capacity was found with increasing Cu concentration compared to reference cultures, except for sublethal levels of Cu in FRAP and CUPRAC tests, where a slight decrease was observed. Under lethal Cu doses, compounds from diatom exudate showed higher DPPH radical inhibition ability than those exuded by control cells (up to 5.43-fold higher), higher Fe(III) to Fe(II) reduction capacity (up to fivefold higher), higher Cu(II) to Cu(I) reduction capacity (up to threefold higher than the control) and much higher Cu-complexation capacity (up to 32.2 times higher).

**Table 4:** Antioxidant activities of *P. tricornutum* exudates cultured under different copper concentrations and collected after 18 days.

Antioxidant assay (units)	Control	Cu(II) 0.31 µM	Cu(II) 0.79 µM	Cu(II) 1.57 µM
DPPH (fmol DPPH cell^-1^)	1.00 ± 0.02	1.12 ± 0.02	4.04 ± 0.00	5.43 ± 0.00
FRAP (fmol Fe(II) cell^-1^)	1.64 ± 0.05	1.53 ± 0.09	4.24 ± 0.03	5.34 ± 0.13
CUPRAC (fmol TR cell^-1^)	1.35 ± 0.01	1.12 ± 0.01	3.78 ± 0.03	3.28 ± 0.08
CCA (fmol Cu cell^-1^)	0.234 ± 0.006	1.646 ± 0.004	3.228 ± 0.004	7.53 ± 0.02

The results are expressed as fmol cell^−1^ (means ± standard deviations of three measurements).

Significantly positive correlations (*p* < 0.05) were observed between the CCA values and the total polyphenol content (R = 0.9820) and total carbohydrate content (R = 0.9806).

## Discussion

This study focuses on analyzing polyphenols and total carbohydrates produced by *P. tricornutum* cells exposed to sublethal and lethal doses of Cu. The entry of microorganisms into the stationary phase activates the mechanisms to adapt and survive at this stage, reprogramming the gene expression pattern until environmental conditions improve^32^. Therefore, organic metabolites were quantified at the beginning and at the end of this growth phase and compared with those secreted by cells grown in the absence of added Cu^25^. In addition, Rahman et al. reported that *P. tricornutum* showed a higher total phenolic content in the stationary stage than in the exponential growth phase^33^, and a higher carbohydrates accumulation during this growth stage was also exhibited by diatoms and dinoflagellate species exposed to different Cu treatments (from 0.16 to 0.79 μM)^34^. Furthermore, Lombardi et al. found strong copper complexing agents released by freshwater Cyanophyta mainly in the stationary growth phase^35^.

*P. tricornutum* diatoms exposed to 1.57 μM Cu for 18 days exuded total levels of all identified polyphenols up to 18.8-fold higher than those exuded by control cells (Table 2), and reached 11.3-fold higher amounts inside the cell (Table 1 and Fig. 2)^25^. The increased production of polyphenols, well known antioxidants, could be explained by their ability to interrupt radical chain reactions and thus prevent or limit cell damage^29,36^. Our results agree with those reported by López et al.^10^, who found that marine microalgae *Dunaliella tertiolecta* grown in the presence of high levels of Cu exuded most of the polyphenols into the medium to alleviate Cu toxicity. Li et al.^1^ studied Cu and Zn accumulation and detoxification strategies in the freshwater green microalga *Chlamydomonas reinhardtii* (*C. reinhardtii*), showing that extracellular matter played a major role in Cu sorption and resistance, which was not observed in the Zn tolerance strategy, where extracellular substances played a minor role.

Relevant differences were found between intra- and extracellular phenolic profiles as a function of metal level and culture period, i.e., GAL was below the LOQ in exudates (Table 2), while it was the most abundant phenolic compound in cells, reaching an amount up to 45.8 times higher than in control cells^25^. Moreover, the phenolic amounts released in the Cu sublethal dose experiment were lower than those exuded by control cells, while the content inside the cells in the same experiment increased. Diatoms have multiple mechanisms to fight against metal toxicity inside the cell, such as gene regulation, chelation, transport into compartments, vacuoles or other organelles, causing heavy metal immobilization, etc.^37–40^, but only exuded and cell wall-associated compounds seem to participate in detoxification mechanisms externally^1^. Therefore, the differences observed in phenolic profiles appear to be the cellular response to different requirements for survival inside and outside of Cu-exposed diatoms.

We observed that the amount of extracellular carbohydrates exuded by *P. tricornutum* cells was also strongly affected by the Cu concentration in the medium after 12 and 18 days of culture, increasing their production upon addition of Cu. However, prolonged exposure periods decreased extracellular carbohydrates in control and sublethal Cu level experiments and increased them under lethal conditions (Table 3). These results are consistent with those previously reported by Santiago-Díaz et al. for amino acids^25^, primary metabolites essential in the growth and development functions of microorganisms^41^, which decreased during the growth phase of highest biomass accumulation in control cells and under sublethal Cu concentrations and increased under lethal conditions. Under the latter conditions, cells survive by decreasing various cellular activities, such as growth, and focusing on defense mechanisms^32,42^.

Our study showed that carbohydrates exuded by cells increased up to 30- and 107.4-fold in the medium enriched with lethal Cu concentrations after 18 days compared to the control (Table 3). This increased carbohydrate exudation may reduce Cu ion activities in the surrounding medium and may facilitate Cu detoxification mechanisms by changing its speciation, improving the tolerance of *P. tricornutum* to Cu stress^41,43–45^. In addition, carbohydrates have also been reported to be involved in other metal detoxification mechanisms such as direct quenching of ROS^46^ and biotransformation of Cu(II) into less soluble and less toxic CuS^47^.

Our results agree with those of Tonietto et al., who found that carbohydrates were the main components of the exudates of the cyanobateria *Cylindrospermopsis raciborskii* and were strongly correlated with the ligand concentration for Cu (R = 0.997)^48^, concluding that a high diversity of ligands increases the metal buffering capacity of the medium and thus, enhances the tolerance of the biota to metal toxicity. Li et al. also reported increases in extracellular polysaccharides exuded by *C. reinhardtii* under Cu toxicity conditions^1^, with polysaccharides being the main substances bound to Cu on the cell surface*,* and enhanced sorption of *C. reinhardtii* cells with increasing Cu concentration. In addition, a significant correlation of extracellular polysaccharides, proteins and DNA with Cu accumulation was found. Li et al. suggested increased production and secretion of extracellular substances to form a biofilm to improve Cu removal and tolerance of *C. reinhardtii*^1^. In extreme environments, several microorganisms have been found to survive by forming a network of biofilms mainly composed of exopolysaccharides (EPS) with functional groups such as uronic acids, sulfated units, and phosphates. These EPSs are often polyanionic, and act as ligands towards toxic metals increasing cell tolerance^27,49,50^. The potent antioxidant activity of several polysaccharides has been related to the uronic acid content^51–53^, which has been quantified in EPS produced by *P. tricornutum* diatoms in the range of 1.4–6.3%, depending on the extraction conditions^54^.

The radical scavenging ability of the exuded compounds against DPPH and their FRAP, CUPRAC and CCA capacities increased under lethal Cu concentrations (Table 4). Under these conditions, the increased production of free radicals, corroborated by the higher levels of intracellular MDA detected (Table 1), must be equalized by a similar rate of antioxidant production to neutralize them or repair the damage they cause. Furthermore, the ability of the exuded compounds to complex Cu(II) showed the greatest increase (up to 32.2-fold higher than that found in the control experiment). We hypothesize that this increased copper complexing activity of the exudates could be the cellular response to the excess of free Cu(II) outside the cells, decreasing its concentration. In fact, significant positive correlations (*p* < 0.05) were found between the exuded carbohydrates and the total content of the identified polyphenols with CCA values, indicating that both types of metabolites could have an important role in reducing the level of free Cu(II). González-Dávila et al. studied the interaction between exudates released by the marine phytoplankton species *Dunaliella tertiolecta* and copper ions^4^, including adsorption to the cell surface, concluding that exudates are involved in reducing the concentration of free Cu.

No correlation was found between carbohydrate and polyphenol contents and DPPH, CUPRAC and FRAP activities, probably due to the presence of other antioxidants, and the dependency of the antioxidant activities on the assay mechanism/kinetics, the profile of the antioxidants, their structures and mix ratios, and the joint action of the compounds, with either synergistic or additive effects^30,55^. Rahman et al.^33^ reported that carotenoids and phenolics were the major contributors to the antioxidant capacity of *P. tricornutm* cells, which correlated to phenolics in the exponential phase and to carotenoids fucoxanthin and β-carotene in the stationary phase. In any case, a more pronounced antioxidant content/activity (radical inhibition, metal reduction or complexation) associated with higher heavy metal concentrations in the environment has been reported as mechanism providing tolerance to metal ions^38^.

The important role of exudates in Cu accumulation and removal has recently been demonstrated by Li et al.^1^, who found that elimination of these extracellular substances from cultures of *C. reinhardtii* exposed to Cu and Zn intensified metal toxicity and decreased the removal of these two metals, the effect being more pronounced for Cu. In fact, the EC50 (metal concentration (mg/L) required to reduce the cell growth rate by 50%) decreased 28.6 ± 3.4 and 19.1 ± 2.6% for Cu and Zn, respectively, compared to cells with intact exudates. Cu adsorption and its maximum accumulation capacities by *C. reinhardtii* exposed to Cu decreased by 60.0 ± 0.1% and 68.5 ± 0.1%, respectively, when extracellular substances were removed.

Our results agree with previous studies reporting increased contents of intra- and extracellular polyphenols in *P. tricornutum* diatoms exposed to sublethal doses of Cu (0.31 and 0.79 μM with growth inhibitions of 20 and 47.5%, respectively)^20^, suggesting the accumulation of these compounds as a protective action of cells to decline metal toxicity. In addition, accumulation of amino acids and carbohydrates has also been reported as a mechanism of tolerance to elevated levels of heavy metals^49,56,57^. Therefore, we hypothesize that these increases observed in the present study could be a self-defense mechanism to try to minimize the toxic effect of Cu, as well as an adaptation and tolerance strategy of cells^10,42^.

Cu speciation plays an important role in regulating phytoplankton community structure affecting ocean primary production^17^. Vasconcelos et al.^19^ investigated the biological behaviour of the coccolithophore *Emiliania huxleyi* (*E. huxleyi*) grown in seawater enriched with its own exudates and those of *P. tricornutum*, *Porphyra* spp. and *Enteromorpha* spp. and found that *P. tricornutum* exudates induced growth inhibition and stimulated the highest cellular release of Cu-complexing organic ligands of all media (160 ± 5 nM followed by 127 ± 4 nM for *Enteromorpha* spp.; 114 ± 3 and 92 ± 4 nM for *E. huxleyi* and *Porphyra* spp. respectively). They concluded that *P. tricornutum* exudates caused a toxic effect on *E. huxleyi* microalgae.

Cell density strongly influences the toxicity of contaminants as their availability per cell is higher at lower cell density, increasing toxicity^58^. Moreno-Garrido et al.^59^ reported studies focused on Cu growth inhibition tests of four marine microalgal species including *P. tricornutum*, observing that cells cultured at the same Cu concentration accumulated higher amount of metal, and thus showed an increase in toxicity as the initial cell density decreased. The effects of Cu toxicity described here for similar cell densities as those found during phytoplankton blooms (1 × 10^7^ cells L^−1^)^60,61^ could be intensified at lower cell abundances commonly observed in coastal environmental contexts^58,62,63^, where Cu could also be enhanced over the concentrations tested here, up to 3.0 μM^13,15^. Under these conditions, Cu availability per cell remains higher, and phytoplankton could release compounds that inhibit microalgal cell division, modifying microalgal community composition and affecting physiological processes of coastal marine organisms, which could change biodiversity^19^.

Analysis of *P. tricornutum* diatoms grown under Cu stress evidenced a large accumulation and exudation of phenolic and carbohydrate compounds strongly affected by the Cu level in the culture seawater, indicating that these compounds are involved in detoxification mechanisms in the extracellular medium. The antioxidant activities of the exudates corroborated that these diatoms produced relevant amounts of antioxidants in response to metal stress, where the Cu-complexing compounds showed the highest increase. This study helps to partially understand the response of marine diatoms to Cu toxicity through the production of carbohydrates and polyphenols, which may play an important role in regulating the speciation of contaminating trace metals. In addition, the evidenced change in the composition of exudates in the presence of toxicants could cause a toxic effect on coastal phytoplankton communities changing their structure and contributing to the loss of biodiversity.

## Materials and methods

### Chemicals

Methanol (HPLC gradient grade), ethanol and tetrachloroethylene (synthesis grade) were purchased from Scharlab (Barcelona, Spain), D-glucose (Glc), m-hydroxyphenyl, CuSO_4_·7H_2_O, DPPH, 2,4,6-tri(2-pyridyl)-triazine (TPTZ), neocuproine (of reagent grade), pyrocatechol violet (PV) and Trolox (TR) were supplied by Sigma-Aldrich (St. Louis, MO, USA). Formic acid (synthesis grade), Fe_3_Cl·6H_2_O and FeSO_4_·7H_2_O were supplied by Panreac (Barcelona, Spain). Ultrapure water was obtained from a Milli-Q system from Millipore (Bedford, MA, USA).

Polyphenol standards were supplied as follows: gallic acid (GAL), protocatechuic acid (PCA), p-coumaric acid (COU), ferulic acid (FA), catechin (CAT), vanillic acid (VAN), epicatechin (ECAT), syringic acid (SYR) and by Sigma–Aldrich Chemie (Steinheim, Germany); rutin (RU) and gentisic acid (GA) by Merck (Darmstadt, Germany).

### Cultures

Axenic strains of *P. tricornutum* (REC 001B) were provided by the Spanish Bank of Algae (Taliarte, Spain). Microalgae were cultured according to Santiago-Díaz et al. in a clean culture chamber (Friocell FC111) under a complete photoperiod (24 h at 8000 lx)^25^, a temperature of 24 °C and an initial cell density of 1 × 10^7^ cells L^−1^.

Seawater used for cultures was sampled off the coast of Gran Canaria, treated with ultraviolet radiation and passed through 0.45 µm filters. The experimental cultures were carried out with seawater enriched with NO_3_^−^ (883 μM), HPO_4_^2−^ (29.3 μM), and SiO_3_^2−^ (142 μM)^64^. For Cu exposure treatments, seawater was enriched with 0.31 µM, 0.79 µM and 1.57 µM Cu (II). Controls were prepared as described above but without Cu addition. After 12 and 18 days of growth, cells were filtered by gravity to avoid rupture using 1.2 µm filters (trace metal acid clean pore-size nitrocellulose, Sarthorius™). The cell density was calculated daily with a light microscope (Microbiotest, Inc.) equipped with a hemocytometer counter, and spectrophotometrically (USB4000) by measuring the absorbance (Abs) at 670 nm. The effect of Cu toxicity on cell growth, and amino acid and phenolic contents was studied and previously reported^25^. Seawater enriched with exudates produced by these diatoms was also analysed, and the results are summarized in the present study.

### Phenolic compounds identification and quantification

For isolation and concentration of phenolic compounds in cultures of *P. tricornutum,* 700 mL of each sample was passed through SPE cartridges (Macherey–Nagel Chromabond Easy, 500 mg) at a flow rate of 2 mL min^-1^. The retained analytes were eluted with 15 mL of methanol and evaporated to dryness. The residue was resolved in 300 μL of methanol, and the solution was filtered through a 0.22 µm filter to be injected into the HPLC equipment.

The determination of the phenolic compounds by RP-HPLC was carried out with Jasco LC-4000 HPLC equipment provided with a PU-4180 quaternary pump, an AS-4150 autosampler, an MD-4015 photodiode array detector, LC-Net ll interface, a Phenomenex C18 column (250 mm × 4.6 mm, 5 µm) and a Phenomenex guard column maintained at 30ºC. The elution was performed with ultrapure water containing 0.1% formic acid (phase A) and methanol (phase B). The flow rate was 1 mL min^-1^, and the injection volume was 10 µL. The gradient elution method for A was as follows: 0 min, 75%; 30 min, 40%; 40 min, 40%. Finally, the column was washed and reconditioned. Simultaneous monitoring was set at 270 nm (GAL, PCA, CAT, VAN, RU, ECAT, and SYR) and 324 nm (GA, COU, and FA) for quantification^25^. Algae samples were analysed in triplicate and the results were expressed as attomol (amol) cell^-1^.

### Total carbohydrates

The total sugar content in *P. tricornutum* cell cultures was determined following the phenol–sulfuric acid method^26^ with some modifications. One milliliter of seawater was freeze-dried, and the residue was dissolved in 340 μL of distilled water. The resulting solution (100 μL) was mixed with 100 μL of phenol (5%) and 0.9 mL of cold concentrated sulfuric acid (98%), stirred and heated in a water bath at 100 °C for 10 min and cooled in an ice-water bath. The absorbance was measured at 490 nm in a UV–VIS spectrophotometer. The total carbohydrate content was determined from a calibration curve prepared using Glc as a standard (y = 0.0049x + 0.0742; R^2^ = 0.9906), and expressed as picomole (pmol) of Glc equivalent cell^-1^.

### Antioxidant capacity assays

The antioxidant activities of the compounds exuded by cells were evaluated by pre-concentration of 780 mL of seawater enriched with *P. tricornutum* exudates collected after 18 days of culture following the same SPE procedure described in Section “Total carbohydrates”. The analytes were resolved with 300 μL of methanol, and the antioxidant activity assays were performed according to Sethi et al. with modifications^30^.

The radical scavenging activity was evaluated by reaction of 30 μL of sample with 800 μL of DPPH solution (0.044 mM) for 15 min. The absorbance was measured at 515 nm, and the results were obtained from a calibration curve prepared with different concentrations of DPPH (y = 11.987x−0.1352; R^2^ = 0.9996) and expressed as femtomol (fmol) of inhibited DPPH per cell.

The ferric reducing capacity was determined by mixing for 10 min at 37 °C, 10 μL of samples and 1 mL of freshly prepared FRAP-reagent consisting of 100 mL of 0.3 M acetate buffer solution (pH 3.6) with 10 mL of TPTZ (10 mM) in HCl (40 mM), and 10 mL of FeCl_3_·6H_2_O solution (20 mM). The mixture was cooled and the absorbance was read at 593 nm. The results obtained from a standard curve prepared with solutions of FeSO_4_·7H_2_O in distilled water (y = 0.5378x + 0.2275; R^2^ = 0.9969) are expressed as fmol of reduced Fe(III) per cell.

The cupric ion reducing capacity assay was carried out with fresh CUPRAC reagent prepared by mixing equal volumes of CuSO_4_·7H_2_O (10 mM), neocuproine ethanolic solution (7.5 mM) and NH_4_Ac buffer solution (1 M). This reagent (1 mL) was mixed with 20 μL of sample for 30 min. Then, the absorbance was recorded at 450 nm, and the results from a calibration curve prepared with Trolox (TR) (y = 0.2859x + 0.0762; R^2^ = 0.9998) are expressed as fmol of TR per cell.

The Cu(II)-chelating activity was evaluated with PV according to Sánchez-Vioque et al. with modifications^65^. Samples (50 µL) were mixed with 700 µL of sodium acetate buffer (50 mM, pH 6.0) and 20 µL of CuSO_4_ (5 mM) by stirring for 30 min at room temperature. After that, 50 µL of PV (4 mM) was added and the mixture was stirred for 30 min. The absorbance was measured at 632 nm. The results are expressed as fmol of complexed Cu^2+^ per cell.

To measure the statistical relationship between the content of polyphenols and carbohydrates and antioxidant activities, the Pearson correlation test was used. Tests were accepted as statistically significant with *p* values < 0.05.

### Ethical approval

We declare that all ethical guidelines for authors have been followed by all authors.

### Consent to participate

All authors have given their consent to participate in submitting this manuscript to this journal. This research does not involve human participants and/or animals.

## Data Availability

The corresponding author will provide data on request.
